# Microbial Diversity and Correlation between Breast Milk and the Infant Gut

**DOI:** 10.3390/foods12091740

**Published:** 2023-04-22

**Authors:** Kaili Wang, Xiufang Xia, Lina Sun, Hui Wang, Qiu Li, Zhuo Yang, Jing Ren

**Affiliations:** College of Food Science, Northeast Agricultural University, Harbin 150030, China; w17662453075@163.com (K.W.); xxfang524@163.com (X.X.); sunlina@neau.edu.cn (L.S.); huiwang@neau.edu.cn (H.W.);

**Keywords:** breast milk microbiota, infant gut microbiota, diversity, correlation

## Abstract

The gut microbiota is significant for infants to grow and develop in the early stages of life. The breast milk microbiota directly or indirectly influences colonizing and the development of early infant intestinal microbiota. Therefore, we wanted to study the microbial diversity and correlation between breast milk and the infant gut. By sequencing the 16S rRNA V3–V4 regions of microbiome in infant feces 1, 14, 20, 30, and 90 days after delivery as well as those in breast milk using Illumina NovaSeq, we studied the component of microbiome in both human milk and infant stools, analyzed the diversity of microbiota, and explored the relationship between them. We found that the richest bacteria in breast milk were *Acinetobacter*, *Stenotrophomonas*, *Sphingopyxis*, *Pseudomonas*, and *Streptococcus*, with a small amount of *Lactobacillus*, *Bifidobacterium*, and *Klebsiella*. The infant feces were abundant in *Bifidobacterium*, *Escherichia-Shigella*, *Klebsiella*, *Streptococcus*, *Serratia*, *Bacteroides*, and *Lactobacillus*, with a small number of *Acinetobacter* and *Pseudomonas*. *Acinetobacter*, *Bifidobacterium*, *Klebsiella*, and *Lactobacillus* appeared in the breast milk and infant feces, suggesting that they were transferred from the breast milk to the infant’s gut.

## 1. Introduction

At first, human milk was assumed to be germ free; however, with the deepening of research and technological progress, many studies found that in addition to abundant nutrients, breast milk contains numerous microorganisms. Breast milk has a complex microbial composition with a high microbial diversity and large individual differences. Most of the microbiota in breast milk belong to *Proteobacteria* and *Firmicutes* and are mainly *Streptococcus* and *Staphylococcus*. Moreover, typical bacteria from the oral and skin environments are also found in breast milk [1,2]. Breastfeeding has many benefits for newborns, such as reducing the risk of diarrhea and respiratory infections, preventing allergic reactions, and likely preventing chronic diseases [3,4].

The infant gut microbiota is relatively simple at birth and the child acquires a more complicated adult-like microbiota in the first 3 years of life, in which multiple factors influence the gut microbial composition and its dynamic changes [5]. These factors include the type of feeding, use of antibiotics in the perinatal period, and mode of delivery [6,7]. The breast-fed infant intestinal microbiota is mainly composed of *Bifidobacterium* and *Lactobacillus* [8].

Breast milk is one of the earliest fountainheads of microbiota assembly in infants, and the development of the early intestinal microbiota in life affects the maturity of the immune system, the uptake of nutrients, and avoidance of pathogen colonization [9,10,11,12]. The study by Jost et al. [13] found that establishing the neonatal gut microbiota is a critical stage in gut maturation, metabolism, and immune programming, as well as in the short- and long-term health status of the individual. Breast milk beneficially affects this process. Although recent research suggests that microbial exposure exists in the womb and that various bacteria are present in the placenta, amniotic fluid, and meconium [14], breast milk is believed to be the primary source of gut microbiota. Breast milk plays an important part in colonizing the infant microbiota in the early months of life [15]. However, the species of microorganisms that link human milk to the gut are unclear.

The study of Kumar et al. [16] found that geographical location can affect the composition of breast milk, indicating that the lipids and components of human milk microbiota are different in different countries. The study of Lackey et al. [17] has a similar conclusion, suggesting that the bacterial composition of breast milk in the same region is more similar than that in different regions and that sometimes there are different core groups in the same region. For this reason, we chose people from the same region for sample collection in order to reduce the impact of geographical factors.

In this study, we explored the relationship between the bacterial communities of breast milk and infant fecal samples from healthy mothers and their infants by analyzing the microbial diversity in both breast milk and infant stools to provide relevant evidence for wholly describing the breast milk microbiota and to better understand the association between the bacterial communities in human milk and the establishment and development of the microbiota in the infant gut.

Infants are prone to diarrheal disease, lower respiratory tract infections, allergic diseases, obesity, and other diseases that can be significantly reduced by breastfeeding [18,19]. In the present study, we explored the relationship between the bacteria of human milk and those of the infant gut, hoping to find beneficial microbiota in human milk that have a certain regulative effect on infant intestinal microbiota and the intestine barrier and provide the possibility of an infant formula that has similar properties to breast milk so as to prevent related diseases.

## 2. Methods

### 2.1. Participants

The research was mainly organized at Harbin Children’s Hospital and Harbin Nangang Maternity Hospital, China. A total of 15 healthy mothers and their full-term infants participated in the study. Breast milk and infant feces were collected at 1, 14, 20, 30, and 90 days after birth. Three samples were collected on each corresponding day. Inclusive criteria were based on breastfeeding, the absence of metabolic or chronic diseases, and the non-administration of antibiotics or probiotic products. Some clinical data about the mothers were recorded, which included the mother’s age, parity, method of delivery, antibiotic use, and feeding.

### 2.2. Sample Collection

We gathered breast milk from mothers and feces from infants’ nappies when visiting the clinic or conducting the study at home. Mothers washed their hands with soap and cleaned their nipples with disposable cotton swabs to minimize breast milk contamination, then used a manual or electric breast pumping to suck milk and transferred the breast milk using a sterile connector into sterile bottles. Breast milk and fecal samples were frozen instantly at −20 °C and delivered to the laboratory with dry ice for analysis. The remaining breast milk and feces samples were held at −80 °C for more analysis.

### 2.3. DNA Extraction and Illumina Sequencing

To extract DNA from samples, we modified the methods of Lackey et al. [17]. The samples were defrosted at room temperature, 0.2 g of the feces samples and 1 mL of the human milk samples were transferred into sterile tubes. The human milk samples were centrifuged (13,000× *g*) for 10 min at 4 °C and the fat and supernatant layers were removed. The remaining steps were performed using an EZNA^®^ Stool DNA Kit (D4015, Omega, Inc., Norwalk, CT, USA) in accordance with the manufacturer’s instructions, and the quality of the DNA extraction was detected by agarose gel electrophoresis. The DNA was quantified using a UV spectrophotometer. Then, 341F (5′-CCTACGGGNGGCWGCAG-3′) and 805R (5′-GACTACHVGGGTATCTAATCC-3′) were used as primers to amplify the V3–V4 region of the 16S ribosomal RNA (rRNA) gene. The PCR products were confirmed by 2% agarose gel electrophoresis. Genes were sequenced on an Illumina NovaSeq platform, following the standard quality control procedures provided by LC-Bio. Next, paired-end reads were assigned to samples based on their unique barcode and truncated by cutting off the barcode and primer sequences. Paired-end reads were merged using FLASH. Quality filtering on the raw reads was performed under specific filtering conditions to obtain the high-quality and clean tags according to fqtrim (v0.94).

### 2.4. Bioinformatics and Statistical Analysis

Chimeric sequences were filtered using Vsearch software (v2.3.4). Using the differential amplification denoising algorithm (DADA2), we obtained representative sequences with single-base accuracy through steps such as “de duplication” (equivalent to clustering with 100% similarity), and operational taxonomic units (OTU) were constructed using the concept of amplification sequence variables (ASVs). We obtained feature tables and feature sequences and further analysis of diversity, annotation of species classification, and analysis of differences was carried out.

BLAST was used for sequence alignment, and the feature sequences were annotated using the SILVA database for each representative sequence. The R software package was used for producing the plots.

## 3. Results

The mothers’ clinical data were investigated, including their age, parity, mode of delivery, antibiotic intake, and infant feeding situation (Table 1). All mothers were in good health before childbirth.

### 3.1. Sequencing Summary

The total DNA extracted from the 30 human milk and infant feces samples were sequenced by 16S rRNA gene Illumina, which yielded a total of 2,520,825 quality-filtered 16S rRNA gene sequence reads, with a mean (±standard deviation, SD) of 84,028 ± 2089 reads. Following initial processing using the DADA2 workflow, we obtained the feature tables and feature sequences that were used for all further analyses.

### 3.2. Diversity of Breast Milk and Infant Intestinal Microbiota

#### 3.2.1. Cluster Analysis of Human Milk and Infant Feces at the Genus and Species Levels

**Figure 1:**
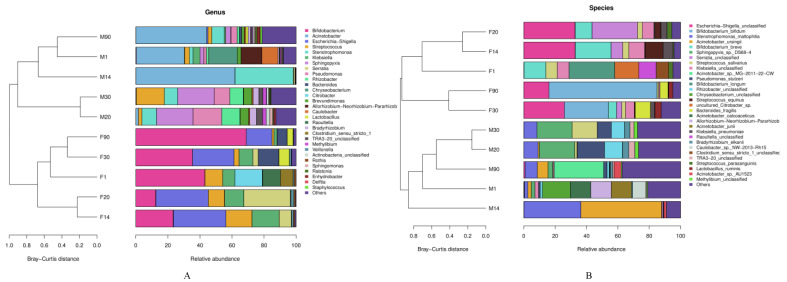
Cluster diagrams of breast milk and infant stools at the level of microbial genera and species. (**A**) The level of microbial genera. (**B**) The level of microbial species. (M: breast milk; F: feces).

Regarding microbial genera, the predominant genus in the mothers’ milk samples was *Acinetobacter*, followed by *Stenotrophomonas* and *Sphingopyxis* (Figure 1A). *Acinetobacter* accounted for 29.95%, 61.74%, and 44.16% of the bacterial genera at 1, 14, and 90 days after delivery, respectively. However, in infant’s fecal samples *Bifidobacterium* was the predominant genus, accounting for 43.06%, 23.29%, 12.37%, 35.35%, and 68.90% at 1, 14, 20, 30, and 90 days after delivery, respectively, followed by *Escherichia-Shigella, Klebsiella,* and *Streptococcus*. In addition, the levels of *Bacteroides* in breast milk samples increased continuously, whereas the levels in infant stools were 12.82% and 6.17% at 30 and 90 days, respectively. The changes in *Bifidobacterium* in the fecal samples of infants generally showed a low–high tendency. The content of *Streptococcus* and that of *Escherichia-Shigella* decreased continuously after 14 days (Figure 1A).

In terms of microbial species, *Stenotrophomonas maltophilia*, *Acinetobacter ursingii*, *Sphingopyxis* sp.-*DS68-4*, and *Acinetobacter* sp.-*MG-2011-22-CW* were predominant in the breast milk samples, which was consistent with the result of microbial genera (Figure 1B). The contents of *Escherichia-Shigella unclassified*, *Bifidobacterium bifidum*, *Bifidobacterium breve*, *Klebsiella unclassified*, and *Bifidobacterium longum* were abundant in the infant fecal samples, although the content of *Klebsiella unclassified* decreased with time. The content of *Bifidobacterium breve* was abundant in the samples at 1, 14, and 20 days, and then decreased continuously. At the same time, the content of *Bifidobacterium longum* was abundant in the 1-day samples and then decreased rapidly. The content of *Bifidobacterium bifidum* decreased with time but then increased continuously after 20 days and was as high as 68.87% at 90 days (Figure 1B).

#### 3.2.2. Heatmap Analysis of Human Milk and Infant Feces at the Genus and Species Levels

**Figure 2:**
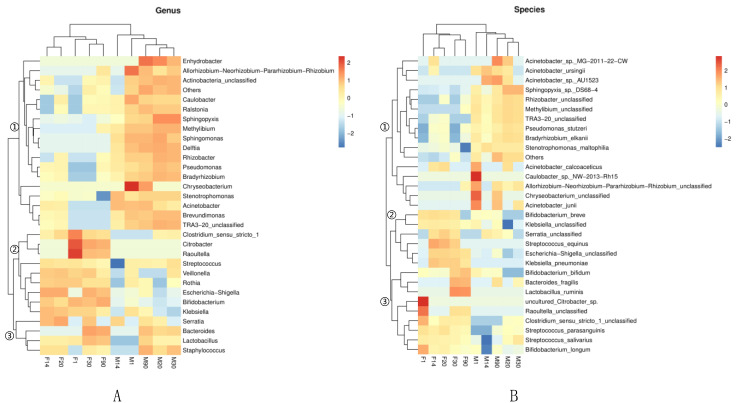
Heatmap of human milk and infant stools at the level of microbial genera and species. (**A**) The level of microbial genera. (**B**) The level of microbial species. (M: breast milk; F: feces; ①, ② and ③ represent three clades ).

Based on the results of the heatmap at the genus and species levels for the microbiota in the milk of the mothers and infant stools, we found that the samples were clustered separately according to their classification. Microbiota in the mothers’ milk and the infants’ stools showed tight clustering, whereas the genera that were abundant in breast milk samples were less distributed in the infant fecal group (Figure 2). However, the genera in clade 3 exhibited a high content in both human milk and infant fecal samples. This clade includes *Bacteroides*, *Lactobacillus*, and *Staphylococcus*, indicating that the microbiota of breast milk is related to the gut microbiota of infants to a certain extent. This finding is the same as the results from Liu Jia and Murphy K et al. [20,21]. At the genus level, *Acinetobacter*, *Sphingopyxis*, and *Stenotrophomonas* were richer in breast milk samples than in fecal samples. The contents of *Stenotrophomonas* increased with time and then decreased after 14 days. By contrast, infant fecal samples contained more *Escherichia-Shigella*, *Bifidobacterium*, and *Klebsiella*. The content of *Bifidobacterium* decreased continuously and then increased with time until it became predominant (Figure 2A).

At the species level, the ratio of *Stenotrophomonas-maltophilia* rose first and then declined continuously after 14 days, whereas the content of *Acinetobacter-ursingii* grew first, then declined to 0.00% at 30 days, and increased to 6.76% at 90 days. At the same time, the content of *Stenotrophomonas-maltophilia* and *Acinetobacter-ursingii* were abundant in the 14-day samples. Moreover, the *Bacteroides-fragilis* content was 9.71% in the 30-day stool sample (Figure 2B).

#### 3.2.3. Bubble Chart Analysis of Human Milk and Infant Feces for Detecting Microbiota

**Figure 3:**
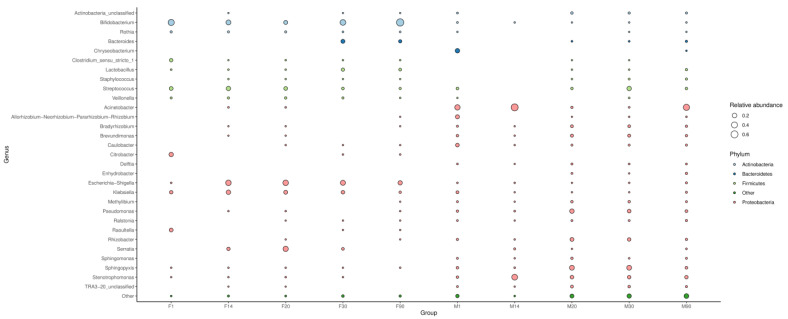
Bubble diagram of microbiota in human milk and infant stools (M: breast milk; F: feces).

The more copious phyla in breast milk samples were *Proteobacteria* and *Firmicutes*, whereas *Proteobacteria* and *Actinobacteria* were predominant in infant feces (Figure 3). This is a little inconsistent with the study by Yassour et al. [22], which reported that *Proteobacteria* and *Actinobacteria* predominated in the infant gut microbial community, whereas *Firmicutes* and *Bacteroidetes* were more abundant in breast milk. The reason may be due to individual differences.

Among *Firmicutes, Streptococcus* was abundant in the milk of the mothers and in infant fecal samples at 1, 14, and 20 days. This phenomenon may be due to colonization into the infant intestine by the relevant bacteria from breast milk. Among *Proteobacteria*, *Acinetobacter* and *Pseudomonas* were found in breast milk samples. The content of *Acinetobacter* increased first, then decreased, and then increased again. The relative richness of *Acinetobacter* was the highest in the 14-day sample, and the *Pseudomonas* content was higher in the 20-day and 30-day samples. However, the contents of *Escherichia-Shigella* and *Klebsiella* among *Proteobacteria* and that of *Bifidobacterium* among *Actinobacteria* were higher in the fecal samples than in breast milk. The content of *Escherichia-Shigella* was low in the 1-day infant fecal sample but was elevated in the 14-day and 20-day samples and decreased afterward. The content of *Bifidobacterium* decreased with time, then increased continuously in the 30- and 90-day samples. This trend is the same as in the studies of Korpela K et al. and Yang R et al. [14,23], who showed that aerobic and facultative bacteria, such as *Streptococcus*, *Enterobacter,* and *Escherichia-Shigella,* had certain advantages in the first three months of life. With the infant growing, the content of oxygen in the gut environment decreases, which is suitable for the growth of *Bifidobacterium* and other anaerobic bacteria, making *Bifidobacterium* the predominant bacterium in the first few weeks of life.

### 3.3. Relationship between Breast Milk and Infant Intestinal Microbiota

#### 3.3.1. Venn Diagram of the Microbiota in Human Milk and Infant Feces

**Figure 4:**
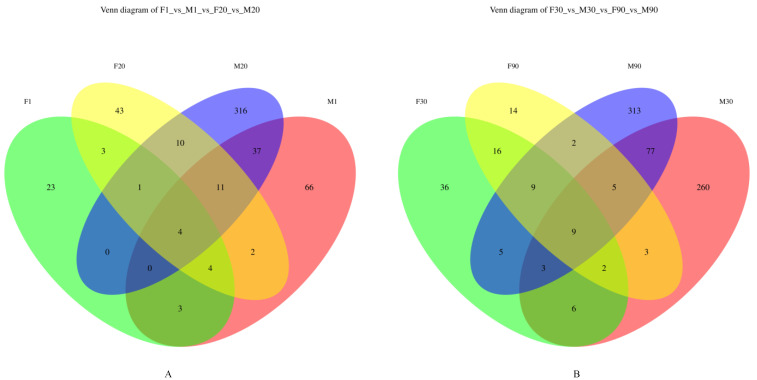
Venn diagrams of microbiota in human milk and infant stools. (**A**) Venn diagram of microbiota in human milk and infant stools on days 1 and 20. (**B**) Venn diagram of microbiota in human milk and infant stools on days 30 and 90. (M: breast milk; F: feces).

Based on the Venn diagrams of the microbiota in human milk and infant stools on days 1 and 20 (Figure 4A) and days 30 and 90 (Figure 4B), we found that the amount of similar operational taxonomic units (OTU) in Figure 4A was four, whereas in Figure 4B it was nine, with 100% similarity. This result suggests that the transfer of the microbiota from the milk of the mother to the infant intestine occurs to a certain extent.

#### 3.3.2. Principal Component Analysis of Microbiota in Human Milk and Infant Fecal Samples

**Figure 5:**
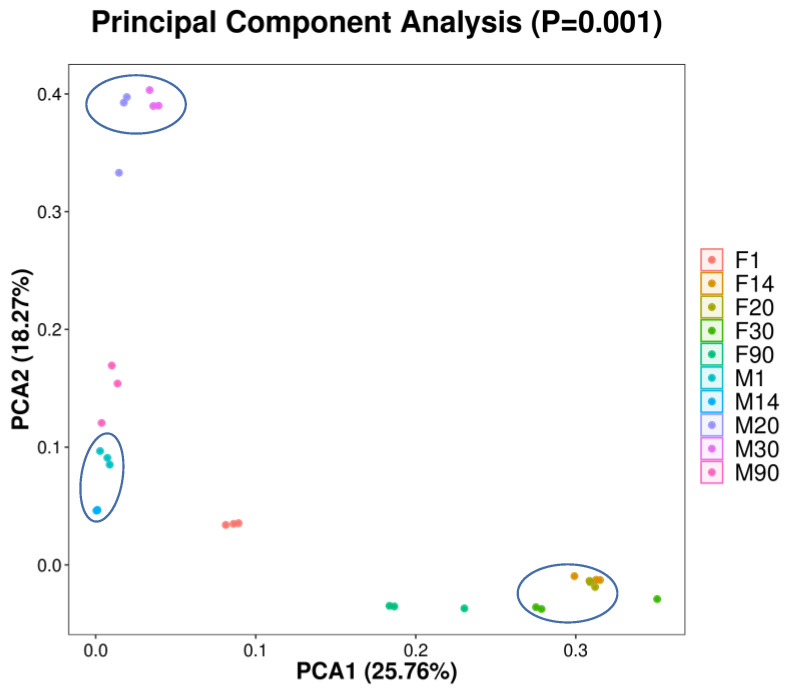
Principal component analysis of microbiota in human milk and infant fecal samples (M: breast milk; F: feces).

We analyzed the differences between human milk and infant gut microbiota using a principal component analysis (PCA) diagram (Figure 5). We found that the microbial component of breast milk samples at 20 and 30 days and at 1 and 14 days was similar. Nevertheless, the microbial distribution of the human milk samples at 90 days was dispersed. The microbial component of the infant fecal samples on different days showed that the distance between the 14-, 20-, and 30-day samples was closer and that the microbial component of the infant stools was closer (i.e., showed higher similarity). In addition, the microbiota in the feces of the 1-day-old infants were clustered together, whereas the microbiota in the fecal samples of 90-day-old infants were dispersed (Figure 5).

#### 3.3.3. Box Diagram of Microbiota in Human Milk and Infant Feces

**Figure 6:**
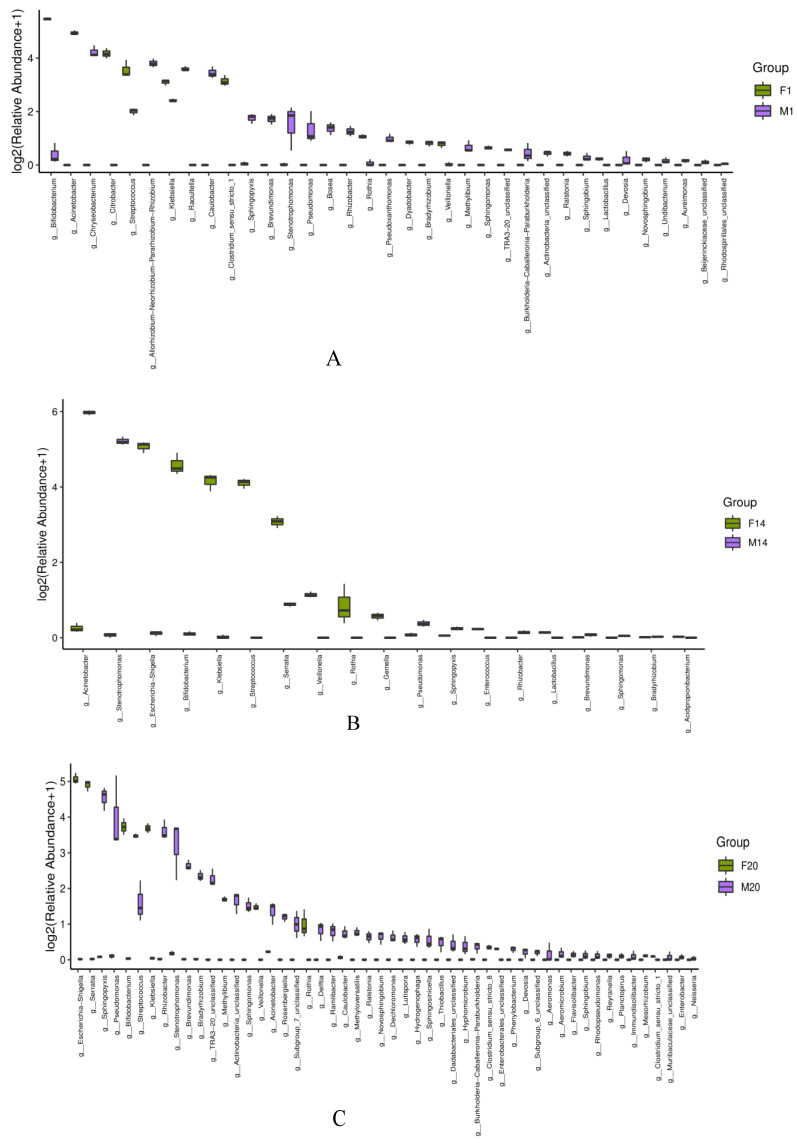

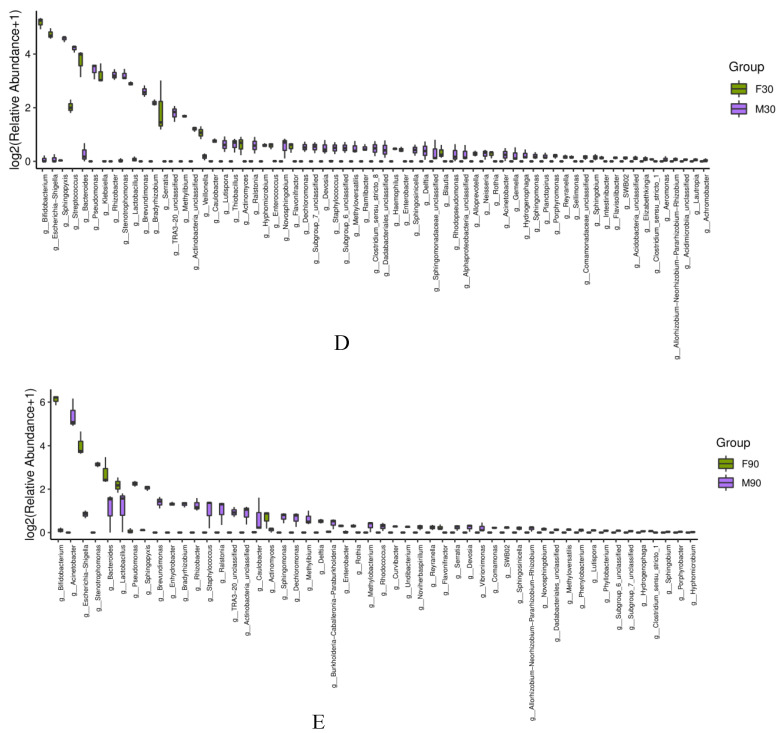
Box charts of microbiota in human milk and infant stools. (**A**–**E**): Box chart of microbiota in human milk and infant stools on days 1, 14, 20, 30, and 90. (M: breast milk; F: feces).

We carried out a similarity comparison between the human milk and infant intestinal microbiota on days 1, 14, 20, 30, and 90 after birth at the genus level. We found that *Bifidobacterium* is more abundant in the fecal samples on day 1, whereas *Acinetobacter* is more abundant in the breast milk samples. The bacteria common to human milk and fecal samples were *Streptococcus*, *Bifidobacterium*, and *Klebsiella* (Figure 6A). *Escherichia-Shigella*, *Bifidobacterium*, and *Klebsiella* were the bacteria with the highest abundances in the fecal samples. The bacteria with the highest contents in the breast milk samples were *Acinetobacter* and *Stenotrophomonas*. *Serratia* existed in both human milk and infant stools. *Acinetobacter* was also found in the infant fecal samples and can be considered to be transmitted from the breast milk to the guts of infants to some extent (Figure 6B). *Escherichia-Shigella*, *Serratia*, and *Bifidobacterium* were the genera with high richness in the infant stools. *Pseudomonas* and *Stenotrophomonas* were the genera with high abundances in the human milk samples. *Streptococcus*, *Stenotrophomonas*, and *Acinetobacter* were the genera common to both samples (Figure 6C).

*Bifidobacterium*, *Escherichia-Shigella*, and *Klebsiella* were the bacteria with high abundances in the infant fecal samples, whereas *Pseudomonas* and *Stenotrophomonas* were the bacteria with high richness in the breast milk samples. *Streptococcus*, *Bacteroides*, and *Lactobacillus* were found in both breast milk and infant stools (Figure 6D). The bacteria with the highest abundances in the fecal samples were *Bifidobacterium* and *Escherichia-Shigella*. The bacteria with the highest abundances in the breast milk samples were *Acinetobacter* and *Staphylococcus*. The genera common to both samples were *Escherichia-Shigella*, *Bacteroides*, and *Lactobacillus*, among them, *Lactobacillus* was more abundant in the infant fecal samples (Figure 6E).

## 4. Discussion

Our results show that the infant intestinal microbiota consisted mainly of *Proteobacteria*, *Actinobacteria*, *Firmicutes*, and *Bacteroidetes*. *Proteobacteria* and *Actinobacteria* were the dominant phyla. *Bifidobacterium* and *Escherichia-Shigella* were more abundant in the infant fecal samples than in breast milk (Figure 1A), which is the same as the findings from Yang et al. [6], who demonstrated that *Bifidobacterium* was the predominant bacteria in the infant gut and had a crucial influence on the growth of the intestinal microbiota during the early days and the subsequent physiological state and health of the child. Nagpal et al. [24] also found that *Enterobacteria* or *Staphylococci* predominated in the day 1 microbiota of healthy infants. However, most of this dominance rapidly transitioned to a *Bifidobacterium*-dominated microbiota within the first few days of life. In addition, we also found that the contents of *Klebsiella-unclassified*, *Escherichia-Shigella-unclassified*, and *Serratia-unclassified* in infant feces increased first and then decreased gradually with time. By contrast, the content of *Lactobacillus* increased gradually with time (Figure 1B), which was similar to the findings of Yang et al. [23], who described that, with the passage of time, beneficial microorganisms such as *Bifidobacterium* and *Lactobacillus* increase in the infant intestine. By contrast, pathogenic bacteria, such as *Escherichia-Shigella* and *Klebsiella*, decrease.

The mother’s milk is considered the basic provenance of bioactive components for the infant. These components may contribute to gastrointestinal colonization, immune development, and maturation of neonates at critical early developmental stages. There are differences in the microbiota components between breast-fed and formula-fed infants, with the former having fewer allergies and gastrointestinal infections [21]. We found that *Acinetobacter*, *Stenotrophomonas,* and *Sphingopyxis* accounted for most of the microbiota in breast milk (Figure 1), which is inconsistent with the results of Lackey et al. [17], who reported that *Staphylococcus* and *Streptococcus* were the core genera of breast milk microbiota. Here, the contents of *Acinetobacter*, *Stenotrophomonas*, and *Sphingopyxis* in breast milk were high, which may be explained by the fact that most mothers in the study delivered their infants by cesarean section. This possibility was also mentioned in the studies by Hermansson et al. and Toscano et al. [25,26], who considered that the method of infant delivery had an standalone effect on the microbial component of human milk. Thus, there are differences that are found in the bacterial compositions of milk from mothers who delivered easily and those who delivered by cesarean section. For instance, the study of Leyva et al. [27] reported the conclusion that *Acinetobacter*, *Streptococcus,* and *Pseudomonas* are common genera in breast milk.

We found that *Acinetobacter* appeared gradually in infant feces. The content of *Bifidobacterium* and *Lactobacillus* in infant feces decreased with time, then increased continuously from day 20. *Acinetobacter*, *Bifidobacterium*, *Klebsiella*, and *Lactobacillus* were all shifted from the human milk to the infant intestine (Figure 6), which proves that human milk is one of the critical fountainheads from the gut microbiota of infants. Our results are in accordance with the discovery of Xi [28] on the correlation between the intestinal flora of newborns and the flora of various parts of the mother’s body. The number of commensal microorganisms that enter the infant’s oral cavity is in the range of 10^−5^–10^−7^ following the ingestion of 800 mL of breast milk per day by the nursing infant. These components of the microbiota include *Acinetobacter*, *Streptococcus*, *Lactobacillus*, *Bifidobacterium*, *Staphylococcus*, *Pseudomonas*, *Klebsiella*, and *Serratia*. They gradually enter the infant’s gut during long-term breastfeeding and play a significant role in building independent intestinal flora in infants.

Numerous studies have shown that the microbiota in human milk can be transmitted to infants and colonize their intestines. The study by Nurrahma et al. [29] found that the mother’s milk is the primary fountainhead for the nutrition for infants and contains many natural probiotics that can be transmitted to infants through it. The bacteria obtained from breast milk included *Enterobacter*, *Staphylococcus*, *Bacteroides*, *Bifidobacterium*, and *Lactobacillus*. Boudry et al. [30] also found a strong overlapping between the infant intestinal microbiota and the breast milk microbiota. The metagenomic analysis conducted by these authors revealed that 76% of the breast milk microbiota species were also found in the infant intestine. The species found in both breast milk and the infant gut included pioneer genera that initiate the fabrication of the intestine microbiota, in particular, facultative anaerobes, such as *Staphylococcus*, *Streptococcus*, and *Lactobacillus*, alongside specialized anaerobes, for instance *Bifidobacterium* and *Bacteroides*.

Breast milk has a crucial influence on establishing the infant gut microbiota by transferring the microorganisms that it contains to the infant’s gut. In this research, some bacteria belonging to different genera but shared by both mothers and infants were identified. The symbiotic and underlying probiotic bacteria in breast milk, including *Streptococcus*, *Bifidobacterium,* and *Lactobacillus* (Figure 6), may be instrumental in the maturity of the intestinal barrier and gut-associated lymphoid tissue. These bacteria may aid in the reduction of chronic illnesses such as diabetes, obesity, and inflammatory bowel disease, as well as the prevention of respiratory and gastrointestinal infections and allergic diseases [31,32]. Similar conclusions were reached by Frank et al. [33], who found that breastfeeding can prevent several acute respiratory and gastrointestinal diseases in some infants before the age of 6 months. Moreover, the future benefits of breastfeeding persist after weaning and can reduce the morbidity of chronic diseases in children.

In addition, some studies have found that human milk oligosaccharides (HMOs), the third most plentiful solid component in breast milk, cannot be absorbed by newborns as nutrients. However, HMOs can promote the growth of instructive microbiota, such as *Bifidobacterium* and some strains of *Bacteroides* and *Lactobacillus* in the infant gut [34,35]. The study by Vandenplas et al. [36] found that, compared with breastfeeding infants, formula-fed infants have higher species richness in infant gut. Human milk can promote the growth of the most balanced microbiota in the infant gut, mainly because it contains large amounts of unique oligosaccharides. Furthermore, HMOs can encourage the growth of specific *Bifidobacteria*. This information suggests that if complete breastfeeding is impossible, the components of infant formulas should be adjusted to promote the growth of gut microbiota dominated by *Bifidobacteria*.

## 5. Conclusions

In summary, we found that the most abundant bacteria in breast milk were *Acinetobacter*, *Stenotrophomonas*, *Sphingopyxis*, *Pseudomonas*, and *Streptococcus*, whereas the most abundant bacteria in infant fecal samples were *Bifidobacterium*, *Klebsiella*, *Streptococcus*, *Bacteroides, Escherichia-Shigella,* and *Lactobacillus*. Among them, *Acinetobacter*, *Bifidobacterium*, *Klebsiella,* and *Lactobacillus* existed in both human milk and infant feces. This finding suggests that the microbiota was transferred from the breast milk to the infant intestine. However, this research lacks more samples and in-depth research on paired individuals; future research is expected to improve the deficiencies.

## Figures and Tables

**Table 1:** Clinical characteristics of the mothers.

Mother’s Serial Number	Sample Days	Mother’s Age	Delivery Times	Delivery Mode	Use of Antibiotics	Infant Feeding Mode
1	1 d	30	Firstborn	Cesarean section	Unused	Breast-fed and formula-fed
2	1 d	32	Firstborn	Cesarean section	Unused	Breast-fed and formula-fed
3	1 d	26	Firstborn	Vaginal delivery	Unused	Breast-fed and formula-fed
4	14 d	34	Firstborn	Cesarean section	Unused	Breast-fed
5	14 d	28	Firstborn	Vaginal delivery	Unused	Breast-fed
6	14 d	27	Firstborn	Cesarean section	Unused	Breast-fed
7	20 d	26	Firstborn	Cesarean section	Unused	Breast-fed
8	20 d	28	Firstborn	Cesarean section	Unused	Breast-fed
9	20 d	29	Firstborn	Cesarean section	Unused	Breast-fed
10	30 d	30	Firstborn	Vaginal delivery	Unused	Breast-fed
11	30 d	31	Firstborn	Cesarean section	Unused	Breast-fed
12	30 d	30	Firstborn	Cesarean section	Unused	Breast-fed
13	90 d	33	Firstborn	Cesarean section	Unused	Breast-fed
14	90 d	29	Firstborn	Cesarean section	Unused	Breast-fed
15	90 d	25	Firstborn	Vaginal delivery	Unused	Breast-fed

## Data Availability

The data used to support the findings of this study can be made available by the corresponding author upon request.
